# Epsom College

**Published:** 1916-06-03

**Authors:** 


### EPSOM COLLEGE.

These are days in which appeals for subscriptions multiply and abound, and members of the medical profession, in addition to certain special claims, share in this respect the fate of their fellow-citizens. No one will propose that though charity is said to begin at home it ought to stay there, and, to take but a single instance, the success of the fund for the Belgian doctors shows that no such narrow view is entertained among British medical men. At the same time it is fair to remark that home claims have a special note of appeal, and it would be a sad thing to see that neglect of these appeared to be a consequence of more remote demands. Both the one and the other need consideration, and if we speak a word here for one of our home institutions this does not mean that we grudge success in other directions. The special and acute emergency speaks for itself, but it ought not to gain a hearing at the expense of a more sustained and continued necessity.

We have before us a brief statement of the immediate position of Epsom College. Many of our readers must know of the high educational standard attained by the College, and of the secure position it occupies among the public schools of the country. But not everyone remembers its close association with the medical profession or is aware of the philanthropic schemes which form an important part of its programme. Attached to the College is a Royal Medical Foundation, and this gives pensions of £30 each to fifty aged members, or widows of members of the medical profession in reduced circumstances, and provides gratuitously an education of the highest class, together with maintenance, clothing, and pocket-money, for fifty necessitous sons of medical men. The Foundation thus does much both to succour those on whom has fallen the stress of misfortune and to secure opportunities for young lives which but for its aid would have no fair outlook.

It is impossible to state a case which would carry on its face a more sympathetic appeal for members of the medical profession. The applicants are always largely in excess of the resources of the Foundation, and it is sad to think of the necessary disappointments which each election inevitably involves. In the present list are the names of at least three boys whose fathers met their deaths in the discharge of professional duty, and it is certain that the losses of medical officers caused by the war will add materially to such claims. The financial position at the moment makes it uncertain whether the scheme can be continued at its existing level, and hence there is the most urgent need for new subscriptions. To contemplate the possibility of narrowed activities carrying messages of consolation and of hope is a painful thing, and we cannot think that the profession will permit it. In every respect Epsom College is worthy of admiration, sympathy, and support. Its Medical Foundation touches both the pathos of misfortune and the promise of the future, and both the one and the other have in themselves as well as in the circumstances of the hour a strong and appealing note.

